# Supplementary material for: Evidence of cooperative effect on the enhanced superconducting transition temperature at the FeSe/SrTiO3 interface
Source: Nat Commun. 2019 Feb 15;10:758. doi: 10.1038/s41467-019-08560-z (PMC6377624; doi:10.1038/s41467-019-08560-z)
Supplement: Supplementary file 1 — Supplementary Information [file 41467_2019_8560_MOESM1_ESM.pdf]

Supplementary Information for

Evidence of cooperative effect on the enhanced superconducting transition temperature at the FeSe/$SrTiO_3$ interface

Song and Yu, *et al.*

Correspondence and requests for materials should be addressed to D.L.F. (dlfeng@fudan.edu.cn) or R.P. (pengrui@fudan.edu.cn)

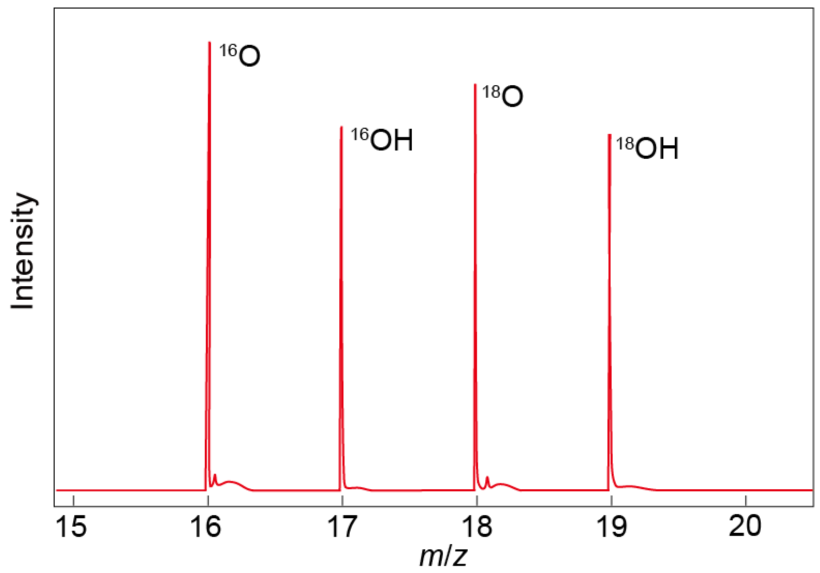

Supplementary Figure 1. Secondary ion mass spectroscopy (SIMS) result. SIMS was performed on a 20nm Se/60-unit-cell $SrTi^{18}O_3$/ $SrTi^{16}O_3$ sample to ensure that the STO films contained a substantial amount of ^{18}O. The result shows that ^{16}O and ^{18}O coexist in our samples, with ^{18}O comprising 47%. This SIMS spectrum was measured when the first >80uc of STO had been sputtered. Since the sample surface is grown and annealed in $^{18}O_2$, and the concentration of ^{16}O diffused from the $SrTi^{16}O_3$ substrate should be less for the surface layers than for the inner layers, the top layer of STO should contain a higher ^{18}O concentration.

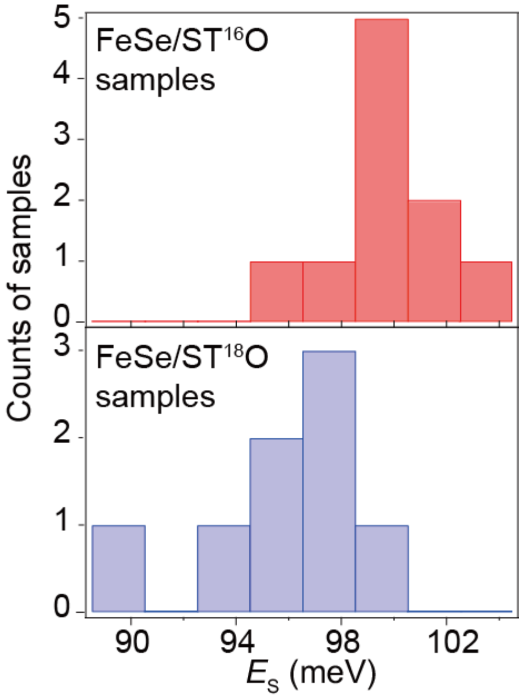

Supplementary Figure 2. Histogram of the energy separations between band γ and γ'. To estimate the deviations from film to film, photoemission data were collected from eighteen samples, and statistical analyses show that the E_S's are 100±2 meV and 95±3 meV for FeSe/ST^{16}O and FeSe/ST^{18}O, respectively.

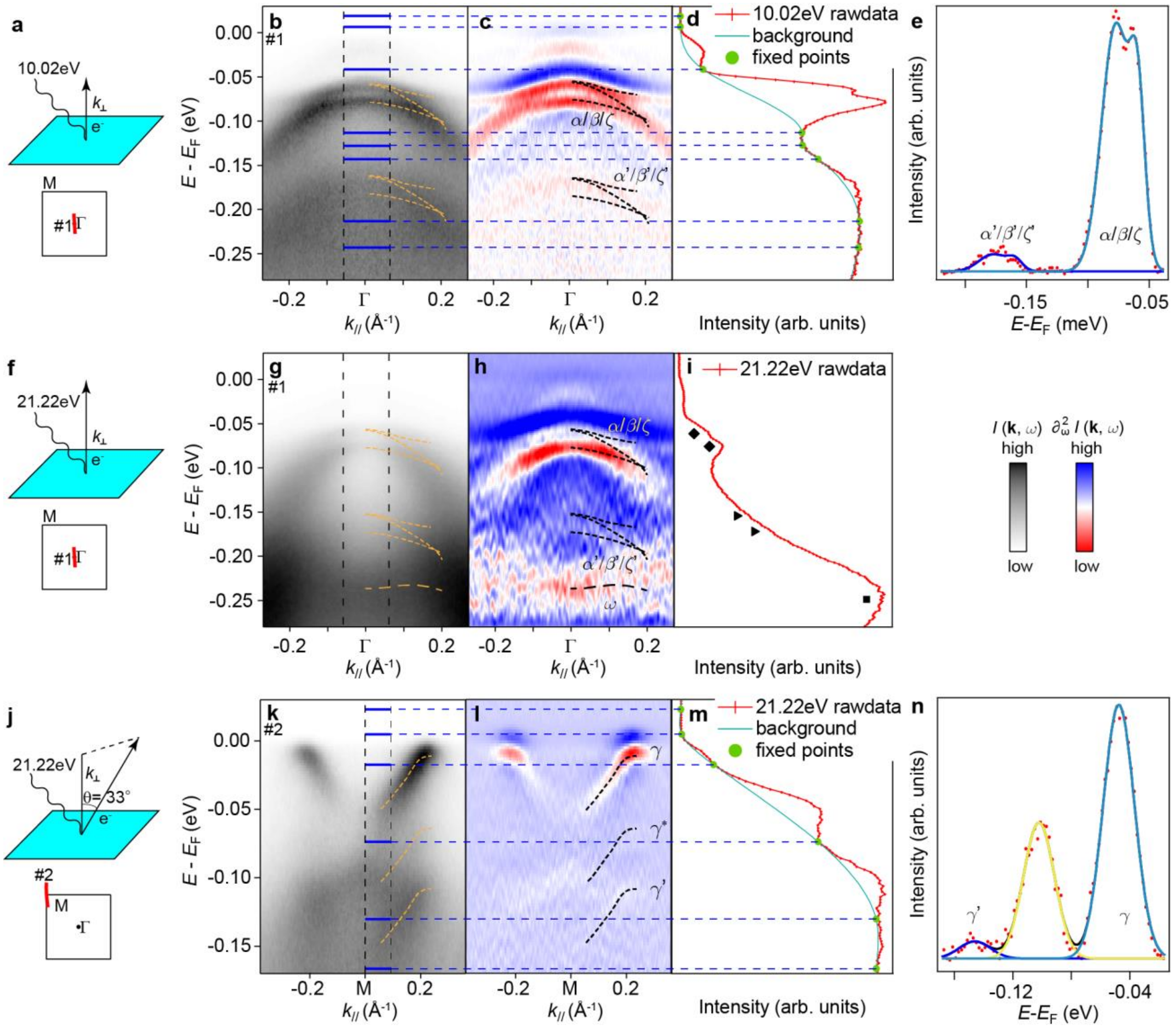

Supplementary Figure 3. Band replica measured using different energies. (**a**) Schematic view of the photoemission geometry with 10.02eV photons (Kr) and normal emission. The corresponding momentum cuts in the first Brillouin zone are illustrated at the bottom of each panel. (**b**) Photoemission intensity along the cut in panel **a** near Γ measured using 10.02eV photons and (**c**) its second derivative with respect to energy. The orange dashed lines on the spectra and black dashed lines on the second derivative spectra indicate the main bands and their replica bands. (**d**) The energy distribution curve (EDC, red curve) integrated over a momentum window between the dashed lines in the panel **b**, and its incoherent background (blue curve) from interpolation of the fixed points (green dots), which corresponds to regions without any main band or replica band features (blue bars in the panel **b**). (**e**) Background-subtracted photoemission intensity data (red dots) and the corresponding fitting results (curves). (**f**) Schematic view of the photoemission geometry with 21.22 eV photons (He Iα) and normal emission. (**g**) Photoemission intensity along the cut in panel **f** near Γ measured using 21.22 eV photons and (**h**) its second derivative with respect to energy. (**i**) The EDC integrated over the momentum window between the dashed lines near Γ in the panel **g**. The diamonds and triangles indicate the main band replica band positions.

(**j**) Schematic view of the photoemission geometry with 21.22 eV photons and 33 °emission angle. (**k-n**) Same as panels **b-e** but along the cut in the panel **j** near M measured using 21.22eV photons.

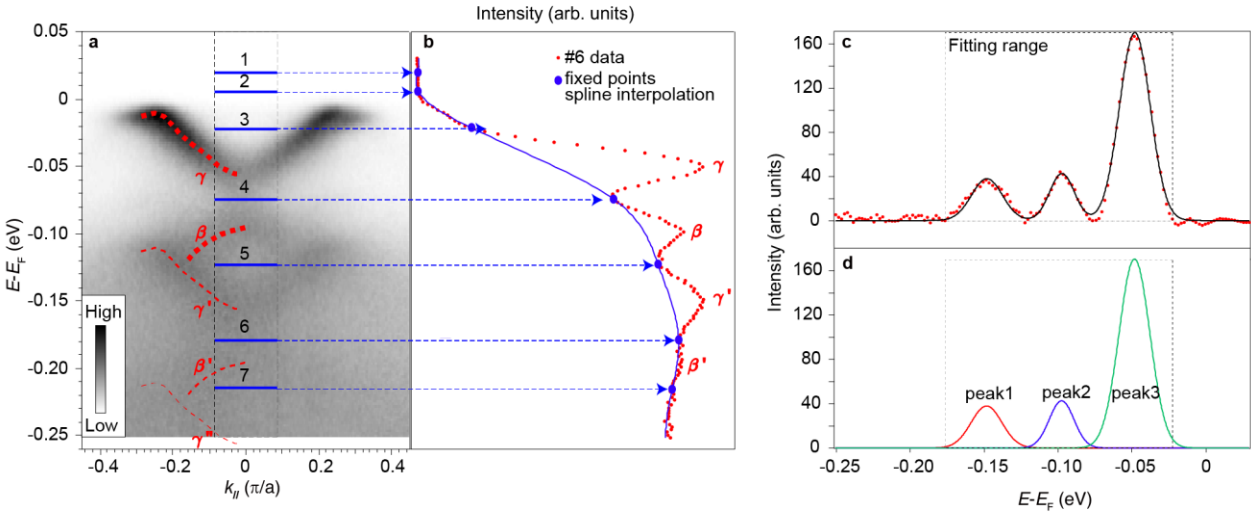

Supplementary Figure 4. Data process of the spectral weight. (**a**) Photoemission spectra around M for sample single-layer FeSe/ST^{16}O #6. The red dashed lines indicate the dispersions of bands γ, β, those of their first order side bands γ', β', and those of their second order side bands γ'', β''. The black dashed rectangle indicates the momentum region Δk where the EDCs of the panel **b** (same as in Fig. 2B of the main text) were integrated. The blue solid lines indicate the energy and momentum regions without any main band or replica band features, and therefore we consider the photoemission intensity integrated at these regions as pure background and use them as fixed points in the interpolation. (**b**) Integrated EDC in the momentum region Δk and the determination of its background by cubic spline interpolation. The red dots indicate the data, and the solid blue circles are the photoemission intensity integrated at the blue line regions in the panel **a**. Using them as fixed points, the background is determined by cubic spline interpolation, following the method in ref. 6. (**c**) and (**d**) Data (dots) and fitting (lines) after background subtraction. The fitting range is fixed at the first three peaks near the Fermi energy which show high signal-to-noise ratios, while the peak representing band β' shows extremely low intensity so that we do not consider it in the fitting. (**c**) Data and total fitting by three Gaussian peaks. (**d**) The three Gaussian peaks plotted separately. The ratio between the density of states of the side band and that of the main band is calculated by the area ratio between peak1 and peak3.

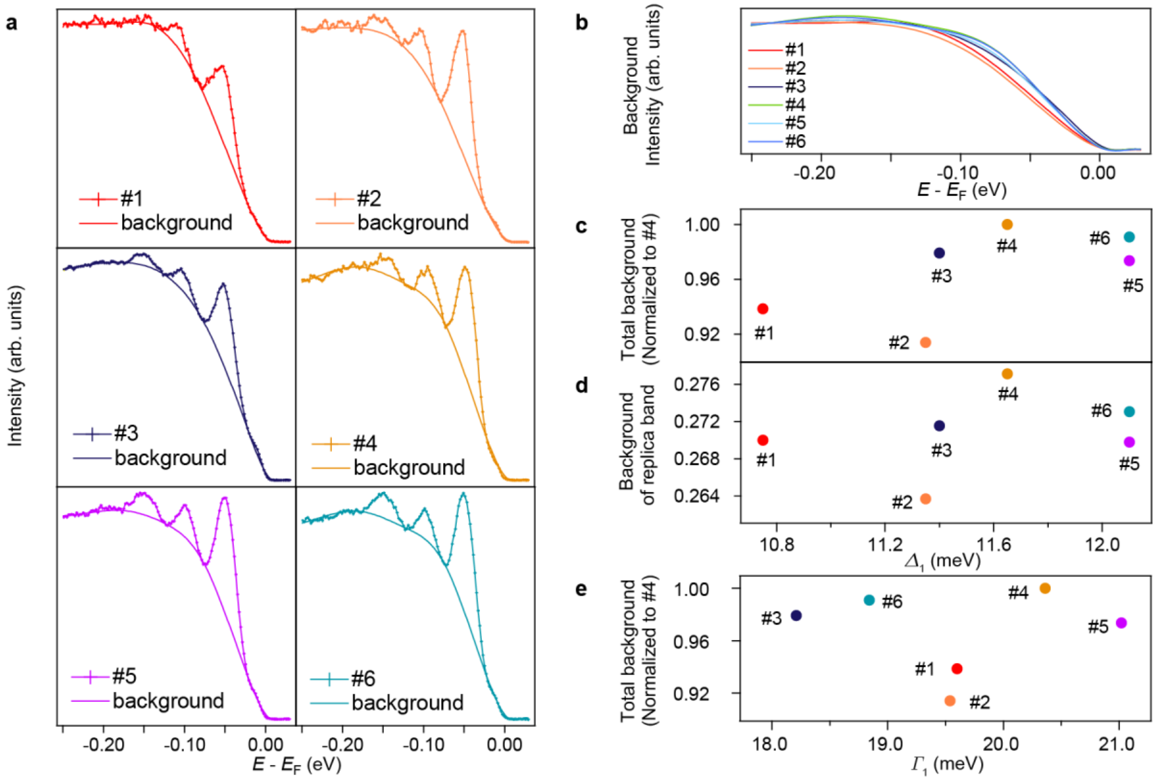

Supplementary Figure 5. Background of samples #1-#6. (**a**) EDCs and the corresponding incoherent backgrounds. (**b**) Comparison of the backgrounds for samples #1-#6. (**c**) The intensity of the total background integrated from *E*=-0.25eV to *E*=0.03eV. The data are normalized to that of sample #4. (**d**) The intensity of background integrated from *E*=-0.17eV to *E*=-0.13eV, the energy range where the spectral weight of the replica band appears. The data are normalized to the total background of sample #4 integrated from *E*=-0.25eV to *E*=0.03eV. (**e**) The relationship between background and superconducting scattering rate (SC peak width).

The background intensity is evidently uncorrelated with the superconducting gap size. The background intensity both over a wide energy range and around the replica band energy (Supplementary Figure 5c and 5d) are random with respect to the superconducting gap size. Also, there is no clear relationship between background and Γ_1, where Γ_1 represents the single-particle scattering rate and is a proxy for FeSe sample quality (Supplementary Figure. 5e), indicating that the variations in background are not induced by defects in the FeSe layer. Supplementary Figure 5e also suggests that the fading of replica band intensity from sample #6 to #1 is not due to defects in the FeSe layer.

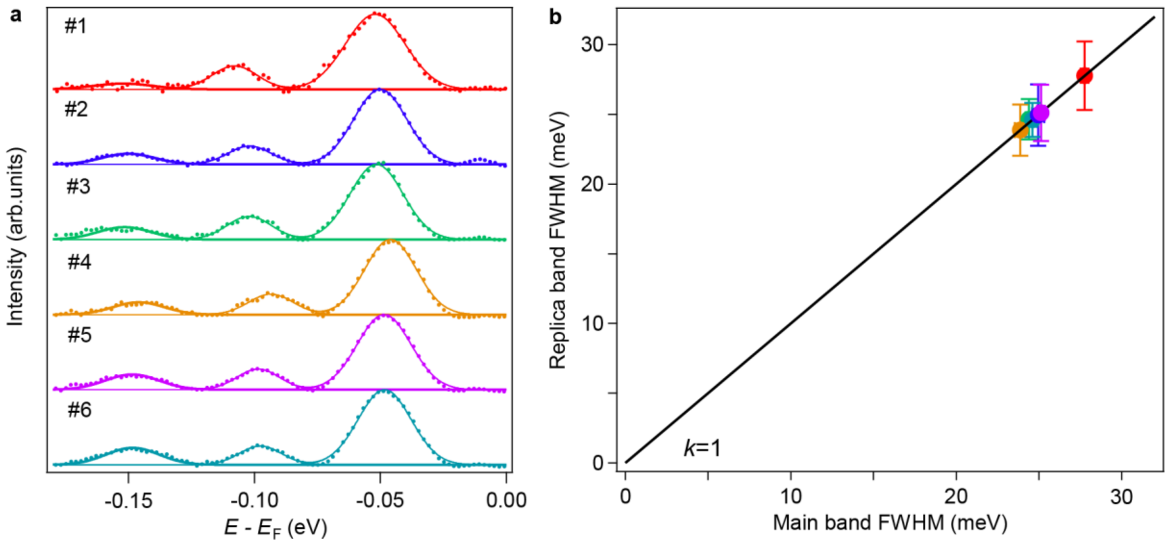

Supplementary Figure 6. The full width at half maximum of the bands. (**a**) Data and fits of the background-subtracted data around the M point of samples #1 ~ #6; (**b**) The relation between the full width at half maximum (FWHM) of the replica band and that of the main band. All data are located along the line y=x, indicating that the FWHM of the replica band's peaks for each sample agrees with that of the corresponding main band's peaks. Uncertainty in the replica band FWHM is taken from the fit's standard deviation.

The fading of spectral weight from sample #6 to sample #1 could be either due to decreased replica band intensity ratio, or due to the smearing out of the replica band. However, if the spectral features of replica bands were smeared out, the replica band would look broader than the main band in samples which have a smaller ratio η, contradicting the observation that the FWHM of the replica band's peak for each sample is identical to that of the corresponding main band's peak.

Therefore, the fading of the replica band intensity from that in #6 sample to that in #1 sample is not due to the smearing out of spectral weight, but is due to the decreasing replica band intensity ratio.

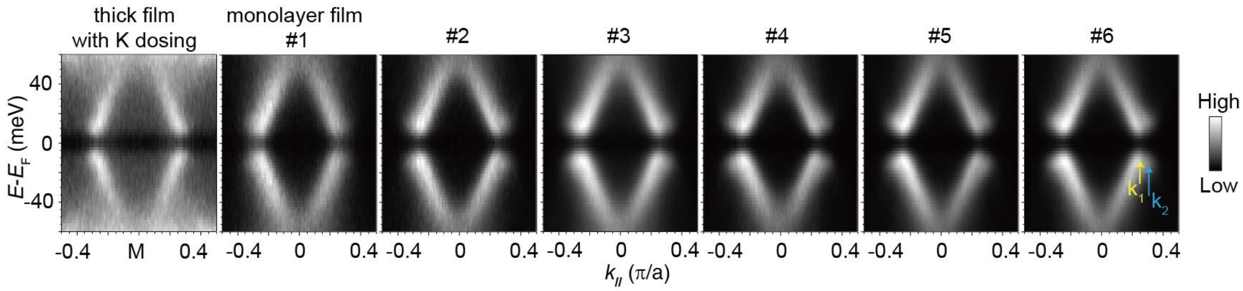

Supplementary Figure 7. Symmetrized photoemission spectra. Symmetrized photoemission spectra across M with respect to the Fermi energy of the representative samples in the main text, including the K-dosed thick film and samples #1-#6. The arrows indicate the normal state Fermi momenta $\mathbf{k}_1$ and $\mathbf{k}_2$.

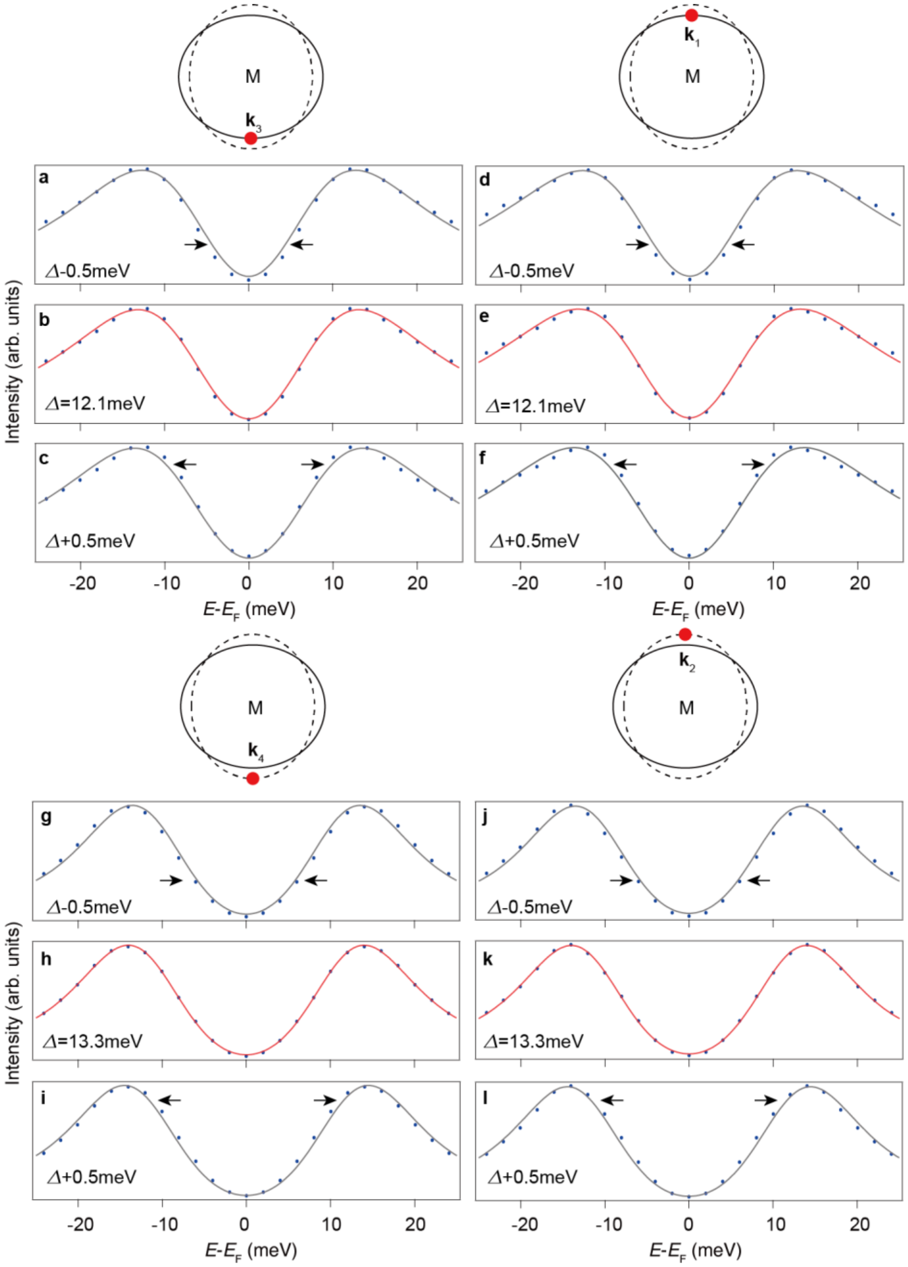

Supplementary Figure 8. Fitting and the energy uncertainty. (**a-c**) Symmetrized EDC data of single-layer FeSe/ST^{16}O #6 at the normal state Fermi momentum $\mathbf{k}_3$ (blue dots), and the fitting to a superconducting spectral function based on the simplified BCS self-energy $\Sigma(\mathbf{k}, \omega) = -i\Gamma_1 + \Delta^2/[\omega + \epsilon(\mathbf{k}) + i\Gamma_0]$, where the Γ_0 was set to 0 in the superconducting state to get the superconducting gap size[2-4]. The fitted gap value Δ is shown in (**b**).

Due to the high sample quality and intense superconducting peak in bands γ and γ', the data near the Fermi energy have very high statistics with extremely low noise. Therefore, fitting such high-quality data can give a gap uncertainty smaller than the energy resolution, i.e., the instrument-induced Gaussian broadening. To estimate the gap uncertainty, we here simulate the spectral function with varied gap size and compare how it deviates from the data. The spectral function is calculated with the gap size manually set to Δ-0.5meV (**a**) or Δ+0.5meV (**c**), respectively. The superconducting spectral function clearly deviates from the measured data (see the arrows

highlighting clear discrepancies between the data and the simulated curves with the manually modified gap sizes). Therefore, ±0.5meV can serve as a conservative estimate of the energy uncertainty of the fitting process. (**d-l**) The same as panels (**a-c**) but at the normal state Fermi momenta $\mathbf{k}_1$ (**d-f**) $\mathbf{k}_4$ (**g-i**), and $\mathbf{k}_2$ (**j-l**). The momentum locations are sketched above each set of EDCs. The gap sizes measured at the equivalent normal state $\mathbf{k}_F$'s of $\mathbf{k}_1$ and $\mathbf{k}_3$ are identical (comparing (**b**) and (**e**), (**h**) and (**k**)). Since the uncertainty of the gap size is ±0.5meV at both $\mathbf{k}_1$ and $\mathbf{k}_3$, their averaged gap size would have an uncertainty of ±0.35meV. Similarly, the uncertainty of the superconducting gap size is ±0.35meV for Δ_2 by averaging the gap sizes measured at $\mathbf{k}_2$ and $\mathbf{k}_4$.

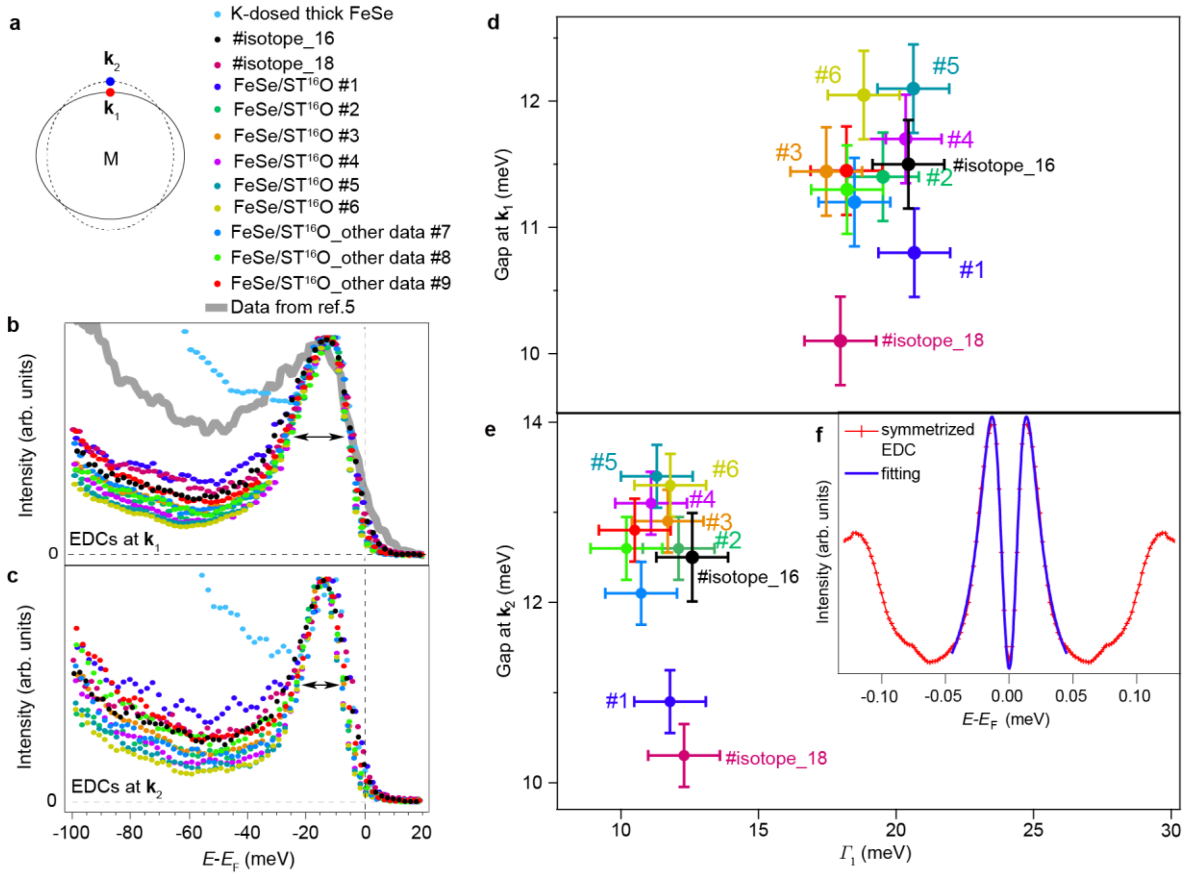

Supplementary Figure 9. Scattering rates of the samples. (**a**) Illustration of the electron pockets, and the Fermi crossings $\mathbf{k}_1$ and $\mathbf{k}_2$. (**b**) EDCs of different samples measured at the normal state Fermi momentum $\mathbf{k}_1$. (**c**) Same as panel (**b**) but at the normal state Fermi momentum $\mathbf{k}_2$. Sample #isotope_16 and #isotope_18 were measured at 12K, data from Ref. [5] were measured at 25K, other samples were measured at 6 K. (**d**) Corresponding gap at $\mathbf{k}_1$ vs. Γ_1. (**e**) Corresponding gap at $\mathbf{k}_2$ vs. Γ_1. (**f**) Example of the numerical fitting, where the red line is the symmetrized EDC and the blue line is the fit curve. K-dosed thick FeSe, and single-layer FeSe/ST^{16}O #1-#6 are the samples used in Figs. 1-4 of the main text. #isotope_16, #isotope_18 and single-layer FeSe/ST^{16}O #7-#9 are used in Fig. 1 and Fig.4 of the main text. The error bar for Γ_1 is due to the finite width of the spectra and system resolution. The error bar for gap size is described in the caption to Supplementary Figure 8.

In all the samples for superconducting gap studies in Figures 2-4 of the main text, the superconducting coherence peaks show almost identical width and similar scattering rates Γ_1 from fitting. The Γ_1's of these samples are significantly smaller than that of the sample used in ref. [5], whose Γ_1 is about 28.3meV (Supplementary Figure 9b), implying the high homogeneity and low defect densities in the current samples. It should be noted that the EDCs at $\mathbf{k}_1$ consist of superconducting coherence peaks of the inner electron pocket and spectral weight from the band dispersion of the outer electron pocket, thus they are broader than those at $\mathbf{k}_2$, which only consist of the superconducting coherence peak of the outer electron pocket. The consistent and superior sample quality ensures that the superconducting gap variation in these samples is not caused by variations in the quality of FeSe. Moreover, there is no correlation between the Γ_1's and

superconducting gaps for these samples. This further excludes the possibility that the gap variation in this work is due to the quality of FeSe.

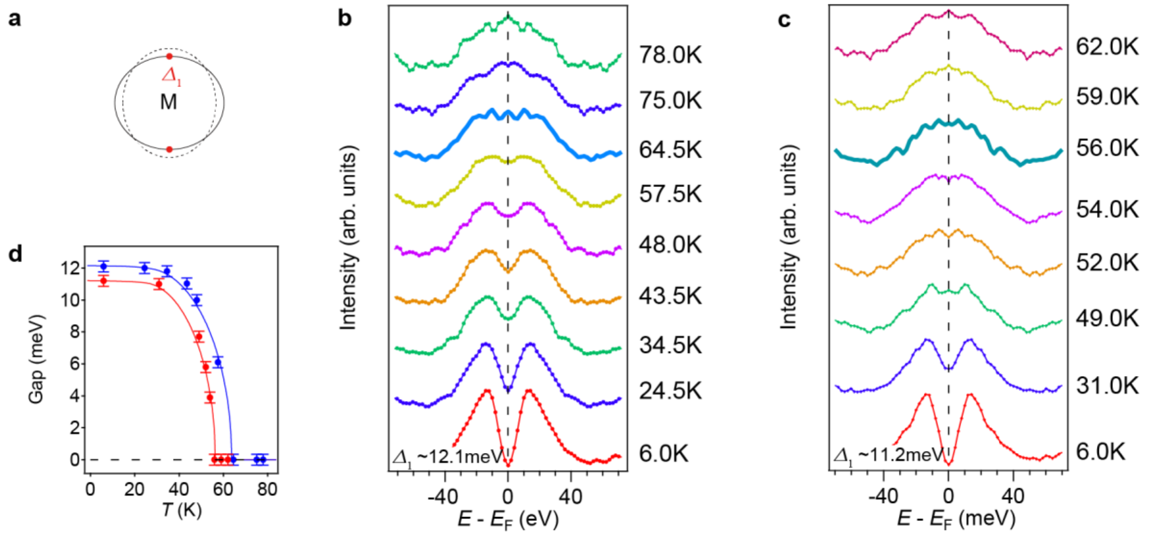

Supplementary Figure 10. Superconducting gap closing temperature. (**a**) Illustration of the Fermi surface around M and the momentum location of $\mathbf{k}_1$ (red dots). The superconducting gap at $\mathbf{k}_1$ is defined as Δ_1. (**b**) and (**c**) Temperature dependence of the symmetrized EDCs for the sample with Δ_1=12.1 meV at 6 K (**b**) and the sample with Δ_1=11.2 meV at 6 K (**c**). The gap decreases with increasing temperature, and eventually closes. (**d**) Plot of superconducting gap versus temperature from data in panels **b** (blue) and **c** (red). The curves are fits to the Bardeen-Cooper-Schrieffer formula. These results give the relation between T_c and the gap size. T_c=64±4 K for the sample with Δ_1=12.1 meV at 6 K, and T_c=56±3 K for the sample with Δ_1=11.2 meV at 6 K. The sample with smaller gap indeed shows a lower T_c. The error bar for gap size is determined as described in the caption to Supplementary Figure 8.

Temperature-dependent ARPES measurements were performed at a limited number of discrete temperatures, so the accuracy in T_c is affected by the step size of temperature variation, which is typically 3K or even larger. Nevertheless, the superconducting gap at 6 K can be precisely determined within an uncertainty of only 0.35 meV in our experiment, which is more reliable than the T_c determined by ARPES to represent the varied superconducting pairing strengths among samples.

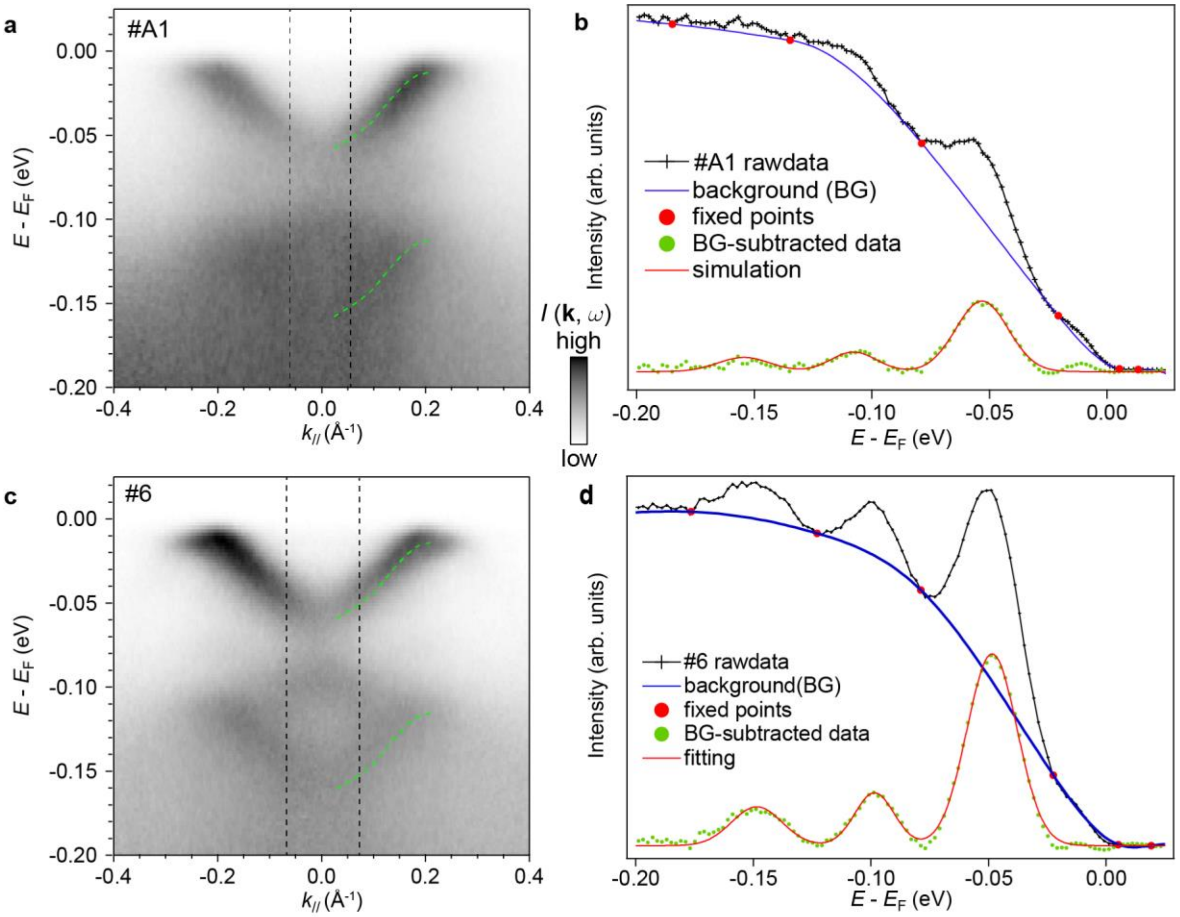

Supplementary Figure 11. Comparison of the data of two samples both with T_c~65 K. (**a**) Photoemission intensity around M for sample #A1, which show T_c~65 K and a higher background. The replica band can be observed, whose dispersion is illustrated by the green dashed lines. (**b**) Corresponding EDC integrated between the black dashed lines on panel **a**, including fixed points, incoherent background, background-subtracted data and simulation. The simulation is performed with $I_1/I_0=\eta=0.22$. (**c**) the same as panel **a**, but for the sample #6. (**d**) the same as panel **b**, except that the red curve is fitting instead of simulation.

The simulation with η=0.22 agrees with the previous data (Supplementary Figure 11b). This confirms that the relation between superconductivity and electron-phonon coupling is robust for both the sample with superior quality (#6) and the previous sample (#A1) with higher background.

On the other hand, although the replica band can be observed in the #A1 sample (Supplementary Figure 11a), both the features of the main band and of the replica band are much less pronounced than the data in #6 (Supplementary Figure 11c), which would significantly affect the accuracy of the quantitative analysis on the replica band intensity ratio if one were to use data from samples like #A1.

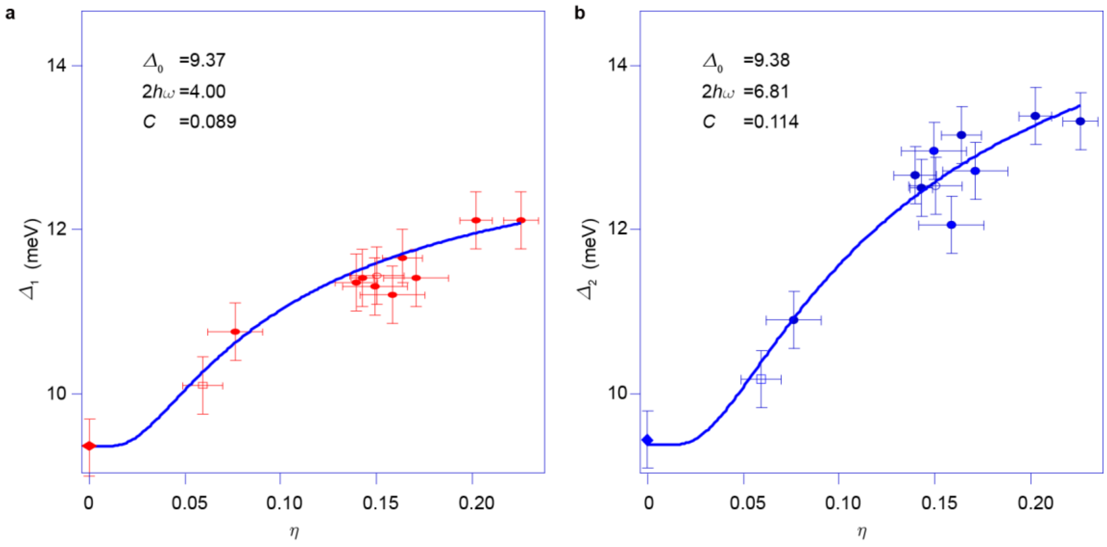

Supplementary Figure 12. Exponential fitting of the gap vs. η. (**a**) Fitting the variation of the superconducting gap Δ_1 with η by the exponential function $\Delta_1 = \Delta_0 + 2\hbar\omega e^{-C/\eta}$. (**b**) Same as panel **a**, but of the superconducting gap Δ_2. The error bar for η is from the standard deviation of the fitting process. The error bar for gap size is described in the caption to Supplementary Figure 8.

BCS theory suggests an exponential relation between superconducting gap size Δ and the electron-phonon coupling through the equation $\Delta = 2\hbar\omega_{\mathrm{D}} e^{-1/N(0)V}$, in which ω_{D} is the Debye frequency, $N(0)$ is the electron density of states at Fermi energy, and V is the electron-phonon pairing interaction.

Our results suggest that the phonon with energy ~94meV is the most effective in enhancing pairing in addition to the intrinsic superconducting mechanism of heavily electron-doped FeSe. If we use the form of the BCS formula for such an interfacial enhancement plus a constant term Δ_0 for other intrinsic paring channels, $\Delta = \Delta_0 + 2\hbar\omega e^{\frac{-C}{\eta}}$ (C is a constant, and η is proportional to V considering the well screened Coulomb repulsion), the fitted $2\hbar\omega$ is 4~6.8meV, which is too small and inconsistent with the phonon energy. Therefore, the exponential fitting is unphysical.

Sample	Growth Temperature measured by pyrometer (℃)	Se/Fe flux ratio	Annealing temperature measured by pyrometer (℃)	Annealing time (hour)
#1	523	9	546	5.5
#2	520	9.1	546	7.5
#3	520	9.3	548	7.5
#4	520	9	547	8.5
#5	520	9	547	5.5
#6	520	9	547	6.5

Supplementary Table 1. Details of the growth and annealing recipes for samples S16 #1-#6.

Note 1. Ruling out the final state shake-off effects as an origin of the replica bands.

The origin of the replica bands has been under intensive debate recently. It was theoretically proposed that the replica band may arise from the coupling between the STO FK phonons and the escaping photoelectron[1], rather than the coupling between FeSe electrons and STO FK phonons. To distinguish these two scenarios, photon-energy-dependent ARPES studies have been performed, in which the perpendicular momentum of the photoelectron is varied (Supplementary Figure 3) and the probability of the proposed photoelectron energy loss can thus be tuned. If the replica band is due to the final state shake-off effect, we can estimate the intensity ratio difference based on the single-loss probability given in Ref. [1]:

$$P(\omega) \propto \frac{1}{k cos\vartheta} \propto \frac{1}{\sqrt{E} cos\vartheta} \qquad (1)$$

Here we compare the band structure around Γ measured using 10.02 eV photons from Kr light (Supplementary Figures 3b-e), and around both Γ (Supplementary Figures 3g-i) and M (Supplementary Figures 3k-n) measured using 21.22 eV He Iα light. Band structure around M measured under 10.02 eV illumination is not included here, because to reach M by ARPES using 10.02eV photons requires an emission angle up to θ=69°, which is beyond the angular range of our apparatus. Moreover, Supplementary Equation 1 is most accurate for emission angles <60 °as suggested in ref. [1].

The replica band intensity could not be quantitatively analyzed in Supplementary Figure 3g because it is buried in the spectral weight tail of the ω band. On the other hand, the band structure around Γ measured using 10.02 eV photons (Supplementary Figures 3b-e) closely resembles that measured using 21.22 eV photons (Supplementary Figures 3g-i), except for the significant intensity suppression of the ω band due to its matrix element effect, which benefits the quantitative analysis.

Comparing Supplementary Figures 3b-d and 3k-m, the replica bands can both be observed although the $k_{\perp}$ of the photoelectron varies. Although the replica bands in Supplementary Figures 3b-d and 3k-m are from different main bands (band $\alpha/\beta/\zeta$ and band γ, respectively), the corresponding escaping photoelectrons are all free electrons, whose probability of suffering a final state shake-off effect should be determined only by $k_{\perp}$ according to Supplementary Equation 1. Quantitative analysis on the replica band ratio is performed below.

Three bands $\alpha/\beta/\zeta$ and their replica bands α'/β'/ζ' are observed around Gamma under 10.02eV, in which two are degenerate in the area between the dashed lines on Supplementary Figure 3b. Thus, two Gaussian peaks are used to fit the main bands and another two are used to fit the replica bands, resulting in $\eta_{\mathrm{Kr}} = 0.069$ (Supplementary Figure 3e). The main band γ and its replica band γ' are observed around M under 21.22 eV. One Gaussian peak is used to fit the spectral weight of the main band and another one is used to fit that of the γ', resulting in $\eta_{\mathrm{He}} = 0.067$ (Supplementary Figure 3n). They are different with a ratio $\frac{\eta_{\mathrm{Kr}}}{\eta_{\mathrm{He}}} = 1.02$.

Considering the work function $\varphi = 4.29eV$, binding energy $E_{\mathrm{BG}} \approx 0.07eV$ at Γ (Supplementary Figures 3b-d) and $E_{\mathrm{BM}} \approx 0.05eV$ at M (Supplementary Figures 3k-m), the photoelectron energy should be $E_{\mathrm{Kr}} \approx 5.66eV$ and $E_{\mathrm{He}} \approx 16.88eV$. The angle of the electron relative to the surface normal is $\theta_{\mathrm{Kr}} = 0°$ for Γ, and $\theta_{\mathrm{He}} = 33°$ for M under 21.22 eV photons, resulting in

$$\frac{\eta_{\mathrm{Kr}}}{\eta_{\mathrm{He}}} = \frac{P_{\mathrm{Kr}}}{P_{\mathrm{He}}} = \frac{\sqrt{E_{\mathrm{He}}}cos\theta_{\mathrm{He}}}{\sqrt{E_{\mathrm{Kr}}}cos\theta_{\mathrm{Kr}}} \approx 1.45$$

which is much larger than the experimental value $\frac{\eta_{\mathrm{Kr}}}{\eta_{\mathrm{He}}} = 1.02$.

The observed $\frac{\eta_{\mathrm{Kr}}}{\eta_{\mathrm{He}}}$ indicates that the momentum of the emitted photoelectron does not play a role in the replica band intensity. This does not agree with the theory that proposes the replica band to be a final-state shake-off effect, while it supports an origin in the coupling between FeSe electrons and STO FK phonons.

Moreover, there are two more facts that cannot be explained by final state shake-off effects:

1. We find that the phonon energy we obtained by EELS (Ω) is different from the energy separation between γ' and $\gamma(E_s)$ obtained by ARPES, due to renormalization by electron-phonon interactions. However, in the process of the final state shake-off effect, the energy loss through phonon excitations of the outgoing electrons should be the same as the phonon energy, in contradiction with our observations.
2. We demonstrate a direct correlation between T_c and the relative intensity of the replica band, a correlation which would not exist if the replica bands were from final-state shake-off effects.

References

1. Li, F. M and Sawatzky, G. A. Electron Phonon Coupling versus Photoelectron Energy Loss at the Origin of Replica Bands in Photoemission of FeSe on SrTiO3. *Phys. Rev. Lett.* **120**, 237001 (2018).
2. Xu, H. C. *et al*. Highly anisotropic and twofold symmetric superconducting gap in nematically ordered $FeSe_{0.93}S_{0.07}$. *Phys. Rev. Lett.* **117**, 157003 (2016).
3. Norman, M. R., Randeria, M, Ding, H., Campuzano, J. C. Phenomenology of the low-energy spectral function in high-T_c superconductors. *Phys. Rev. B* **57,** R11093 (1998).
4. Zhang, Y. *et al*. Nodal superconducting-gap structure in ferropnictide superconductor $BaFe_2(As_{0.7}P_{0.3})_2$. *Nat. Phys.* **8**, 371-375 (2012).
5. Tan, S. Y. *et al*. Interface-induced superconductivity and strain-dependent spin density waves in $FeSe/SrTiO_3$ thin films. *Nat. Mater.* **12**, 634–640 (2013).
6. Lee, J. J. *et al*. Interfacial mode coupling as the origin of the enhancement of T_c in FeSe films on $SrTiO_3$. *Nature* **515**, 245–248 (2014).
